# Clinical and radiological features and skeletal sequelae in childhood intra-/juxta-articular versus extra-articular osteoid osteoma

**DOI:** 10.1186/s12891-015-0456-y

**Published:** 2015-01-31

**Authors:** Mi Hyun Song, Won Joon Yoo, Tae-Joon Cho, Chin Youb Chung, Moon Seok Park, Jung-Eun Cheon, In Ho Choi

**Affiliations:** Department of Orthopedic Surgery, Jeju National University Hospital, Jeju, South Korea; Department of Orthopedic Surgery, Seoul National University Children’s Hospital, 101 Daehak-ro, Jongno-gu, 110-744 Seoul, South Korea; Department of Orthopedic Surgery, Seoul National University Bundang Hospital, 82 Gumi-ro, 173 Beon-gil, Bundang-gu, Kyunggi-do 463-707 South Korea; Department of Pediatric Radiology, Seoul National University Children’s Hospital, 101 Daehak-ro, Jongno-gu, 110-744 Seoul, South Korea

**Keywords:** Osteoid osteoma, Clinical feature, Skeletal complication, Children

## Abstract

**Background:**

To compare the clinical and radiological features of intra-/juxta-articular osteoid osteoma and extra-articular osteoid osteoma in skeletally immature patients, paying special attention to the skeletal complications.

**Methods:**

Osteoid osteoma in 34 children (22 boys and 12 girls, mean age 10.4 years) was dichotomized according to the location of the nidus as intra-/juxta-articular (11 children) or extra-articular (23 children). The following features were compared: diagnostic delay, typical symptoms, synovitis and limited range of joint motion, response to treatment, typical radiographic findings, and skeletal complications.

**Results:**

Eight of the 11 children with intra-/juxta-articular osteoid osteoma presented with synovitis in the involved joint, which led to a delayed diagnosis for a median 9.5 months. Pain disappeared in all children with surgical or medical interventions, but at the mean 4.9-year follow-up evaluation, skeletal abnormalities around the joint were noted in 5 children (4 proximal femur and 1 distal humerus) with intra-/juxta-articular osteoid osteoma, 2 of whom required subsequent surgeries for limited hip motion caused by femoroacetabular impingement and limited range of elbow motion, respectively. In contrast, typical clinical and radiological features were observed more often in extra-articular osteoid osteoma, and only 1 child showed overgrowth of the tibia, which did not have clinical significance.

**Conclusions:**

Intra-/juxta-articular osteoid osteomas in growing children exhibit different clinical and radiological features from extra-articular lesions. Skeletal abnormalities mainly develop in intra-/juxta-articular osteoid osteoma, and these may lead to permanent skeletal sequelae.

## Background

Osteoid osteoma is a common benign tumor that is usually located in the metaphyseal and diaphyseal regions of long bone [1]. Radiologically, it appears as a radiolucent or partially calcified nidus surrounded by a sclerotic margin [2]. Nocturnal pain markedly responsive to nonsteroidal anti-inflammatory drugs (NSAIDs) is a well-known classic symptom [1]. The goal of treatment is to remove the nidus either by surgical resection or by radiofrequency ablation, but the tumor sometimes spontaneously regresses with NSAID medication alone [3]. When an osteoid osteoma occurs in the joint (intra-articular) or near the joint capsule (juxta-articular), however, the patients may present with unusual clinical and radiological signs that are different from those manifesting in an extra-articular osteoid osteoma [4,5]. In growing children, osteoid osteoma may cause skeletal abnormalities, including overgrowth and angular deformity of the long bone, hypertrophy of the femoral neck with or without increased neck-shaft angle, and increased femoral antetorsion [6-12].

Given the different clinical and radiological features of osteoid osteoma depending on the location of the nidus, and the potential skeletal complications in growing children, a study comparing the two types of osteoid osteomas exclusively in skeletally immature patients is warranted. However, there has been no such study in the literature, and only a few reports provide fragmentary information on skeletal abnormalities in children with osteoid osteoma [6-12]. In the current study, we compared the clinical and radiological features of intra-/juxta-articular osteoid osteoma and extra-articular osteoid osteoma in growing children, paying special attention to the skeletal complications. We hypothesized that skeletal abnormalities develop more frequently in intra-/juxta-articular osteoid osteoma, and these may cause permanent sequelae in skeletally immature patients.

## Methods

This study was approved by the SNUMC/SNUH IRB issued by the Seoul National University College of Medicine and Seoul National University Hospital. The study design was a retrospective case series. We reviewed the medical records and imaging studies of all patients treated for osteoid osteoma in our hospital between July 2001 and April 2012. Patients were excluded from the study (1) when the age at diagnosis was 16 years or older for boys and 14 years or older for girls, (2) when the patients were referred to our hospital after previous treatment that might have altered their clinical features, and (3) when the follow-up period was less than 2 years. Thirty-four children (22 boys and 12 girls) were selected (Table 1). The mean age at diagnosis was 10.6 years (range, 4.2 to 15.9 years) for boys and 10.0 years (range, 6.2 to 13.5 years) for girls. The mean follow-up period was 4.9 years (range, 2.0 to 11.8).

**Table 1:** **Demographic characteristics of the intra-/juxta-articular osteoid osteoma and extra-articular osteoid osteoma**

**Variables**		**Intra-/juxta-articular (n = 11)**	**Extra-articular (n = 23)**	***p*** **-value**
Sex (F : M)		6 : 5	16 : 7	0.459^*^
Age at diagnosis (yr)		10.4 (4.1 to 14.8)	10.4 (4.3 to 15.9)	0.740^**^
Follow-up period (yr)		4.7 (2.0 to 8.1)	5.0 (2.4 to 11.8)	0.619^**^
Treatment method	RFA	5	11	
	Resection	3	7	1.000^*^
	NSAIDs	3	5	
Diagnostic study	Plain radiography	11	23	
	CT	9	12	
	MRI	10	5	
	Bone scan	2	8	

RFA, radiofrequency ablation; NSAIDs, non-steroidal anti-inflammatory drugs. Data presented as number of patients for sex, diagnostic study and treatment methods, and data presented as median (range) for age at diagnosis and follow-up period. *Fisher’s exact test, **Mann–Whitney test.

Thirty-four children were dichotomized according to the location of the nidus as intra-/juxta-articular (11 children) or extra-articular (23 children) (Table 1). Clinical features were investigated with respect to the following: diagnostic delay; presence or absence of the classic symptoms of osteoid osteoma, such as nocturnal pain and the efficacy of NSAIDs in the relief of pain; presence or absence of synovitis and limited range of joint motion; response to treatment; and complications. The radiological features were investigated with respect to the presence or absence of a typical radiolucent nidus with a surrounding sclerotic rim on plain radiographs and skeletal abnormalities, such as widening of the femoral neck, shortening of the femoral neck, coxa valga, bowing of long bone, overgrowth of long bone (>10 mm), and hypertrophy of the bone and cartilage around the joint. Clinical and radiological features were compared between the two types of osteoid osteoma. For statistical analysis, Fisher’s exact test was used to analyze the contingency table, and the Mann–Whitney test was used for a comparison of continuous variables. All statistical analyses were conducted using SPSS version 21.0 (SPSS, Chicago, IL, USA).

## Results

Clinical and radiological features of the 34 patients are summarized in Table 2. Intra-/juxta-articular osteoid osteoma occurred primarily in the proximal femur (n = 8), but also in the proximal humerus (n = 1), distal humerus (n = 1), and calcaneus (n = 1). Extra-articular osteoid osteoma occurred in the femur (n = 12), tibia (n = 8), humerus (n = 1), distal phalanx of the toe (n = 1), and vertebra (n = 1). In intra-/juxta-articular osteoid osteoma, all the niduses were located in the cortex. In extra-articular osteoid osteoma, the niduses were located in the cortex (n = 19), medullary canal (n = 2), and subperiosteal region (n = 2). There was no case of multifocal osteoid osteoma. Four of the 11 children (36.4%) with intra-/juxta-articular osteoid osteoma had nocturnal pain that was relieved by NSAIDs, and this typical clinical feature was observed more frequently in the children with extra-articular osteoid osteoma (16/23, 69.6%), although the difference was not statistically significant (*p* = 0.135). Painful limitation of joint motion was observed in 8 of 11 children (72.7%) with intra-/juxta-articular osteoid osteoma, but this clinical sign of synovitis was not present in any children with extra-articular osteoid osteoma (*p* < 0.001). Seven of 11 children (63.6%) with intra-/juxta-articular osteoid osteoma were initially misdiagnosed with chronic osteomyelitis (n = 2), muscle sprain (n = 2), nondisplaced fracture (n = 1), pigmented villonodular synovitis (n = 1), or periosteal hemangioma (n = 1), and the median duration of the diagnostic delay in these children was 12 months (range, 1.5 to 18 months). In contrast, only 5 of 23 children (21.3%) with extra-articular osteoid osteoma were initially misdiagnosed with chronic osteomyelitis (n = 2), muscle strain (n = 1), juvenile rheumatoid arthritis (n = 1), or growing pains (n = 1) with a median diagnostic delay of 1.5 months (range, 1 to 6 months). The chance of misdiagnosis and diagnostic delay was significantly different between the 2 types of osteoid osteoma (*p* = 0.026 and *p* = 0.002, respectively). On plain radiographs, a typical radiolucent nidus with surrounding sclerotic rim was observed in 4 of 11 children (36.4%) with intra-/juxta-articular osteoid osteoma, and this radiographic finding was observed more frequently in the children with extra-articular osteoid osteoma (18/23, 78.3%) (*p* = 0.026).

**Table 2:** **Comparison of clinical and radiological features between the two types of osteoid osteoma**

**Variables**	**Intra- /juxta-articular (n = 11)**	**Extra-articular (n = 23)**	***p*** **-value**
Classic symptom	4	16	0.135^*^
Synovitis	8	0	<0.001^*^
Misdiagnosis	7	5	0.026^*^
Typical nidus	4	18	0.026^*^
Skeletal abnormality	5	1	0.008^*^
Diagnostic delay (months)	12 (1.5 to 18)	1.5 (1 to 6)	0.002^**^

Data presented as number of patients who had each clinical and radiologic feature, except diagnostic delay in which data presented as median (range). *Fisher’s exact test, **Mann–Whitney test.

**Figure 1:**
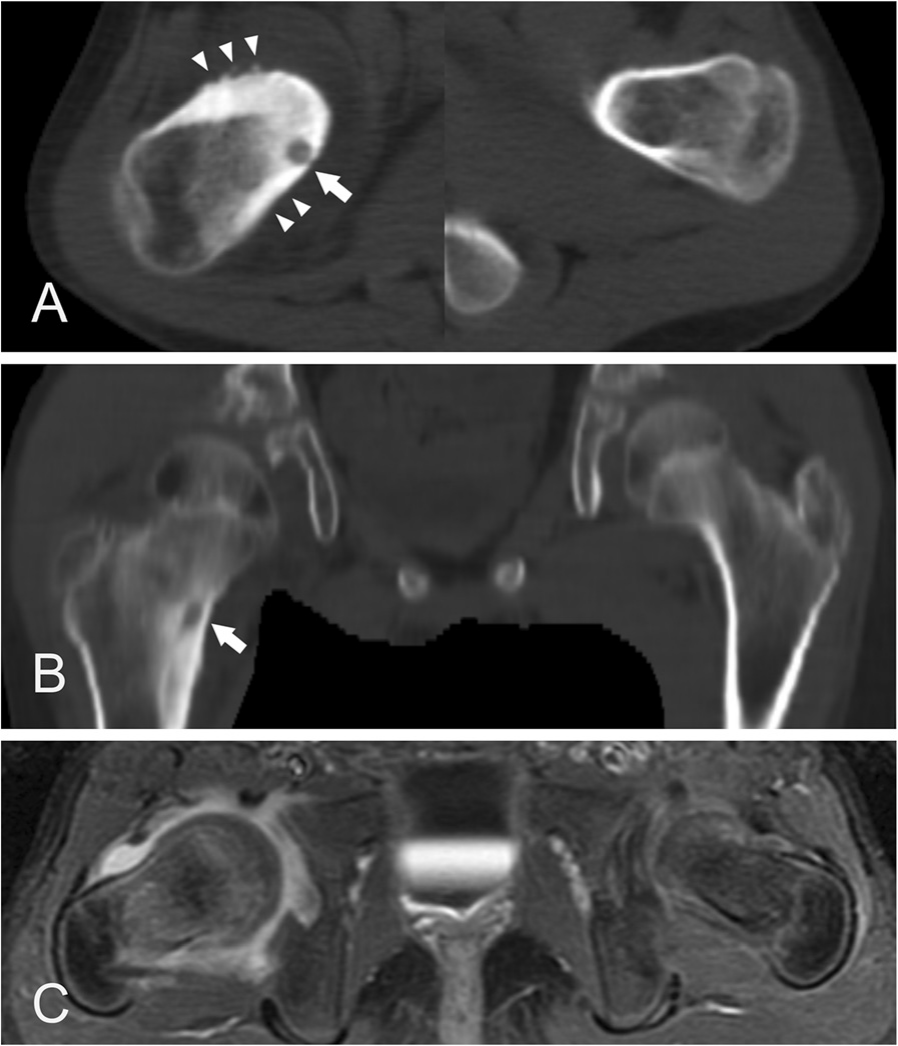
**A 9.7-year-old girl with a juxta-articular osteoid osteoma of the proximal femur with involvement of the hip joint. (A)** Axial CT image of the right femoral neck shows a prominent bony hypertrophy (arrowheads) around the nidus (arrow). **(B)** Coronal CT image demonstrates a nidus just above the lesser trochanter of the femur (arrow). Note the widening of the femoral neck and hypertrophy of the femoral head compared with the contralateral normal side. **(C)** Axial contrast-enhanced T1-weighted MR image with fat suppression shows synovial hypertrophy with effusion. The head-neck offset is not clear.

**Figure 2:**
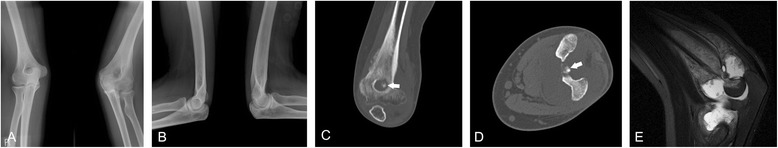
**A 14.1-year-old boy with an intra-articular osteoid osteoma of the olecranon fossa with involvement of the elbow joint. (A, B)** Anteroposterior **(A)** and lateral **(B)** radiographs show hypertrophic deformation around the left elbow joint. **(C, D)** Coronal **(C)** and axial **(D)** CT images demonstrate the nidus (arrow) at the olecranon fossa. **(E)** Sagittal T2-weighted MR image with fat suppression shows a heterogeneous intermediate signal intensity of distal humerus with a high signal intensity around the elbow joint indicating bone marrow edema with synovitis.

Pain was relieved in all patients after radiofrequency ablation of the nidus in 16 children, after surgical removal of the nidus in 10 children, and by NSAID medication for a median of 4 months (range, 3 to 7 months) in 8 children. However, skeletal abnormalities developed in 5 of 11 children (45.6%) with intra-/juxta-articular osteoid osteoma and in 1 of 23 children (4.3%) with extra-articular osteoid osteoma (*p* = 0.008). The skeletal abnormalities observed in the intra-/juxta-articular osteoid osteoma cases were widening of the femoral neck associated with coxa valga deformity (n = 3) or with overgrowth of the femur by 11 mm (n = 1) and hypertrophic deformation of the elbow (n = 1). In contrast, only 1 child with extra-articular osteoid osteoma showed overgrowth of the tibia by 10 mm. Hypertrophy of the toe was observed in 1 child with extra-articular osteoid osteoma in the distal phalanx with slight over growth (2 mm) of the distal phalanx. As the overgrowth was minimal, this was not considered a skeletal abnormality. Widening of the femoral neck did not cause any symptoms in 3 children with intra-/juxta-articular osteoid osteoma, but the remaining child, who was 9.7 years old, had been misdiagnosed as having pigmented villonodular synovitis for 13 months and suffered persistent limitation in hip flexion and internal rotation even after pain relief with NSAID treatment. In this child, there was a cam-type of femoroacetabular impingement with marked hypertrophy in the epiphysis as well as in the metaphysis of the proximal femur. She underwent osteochondroplasty to create a head-neck offset using the surgical hip dislocation approach. She acquired full range of hip motion after surgery (Figure 1). The other case with symptomatic skeletal complications was a child who had intra-articular osteoid osteoma in the olecranon fossa. This child was misdiagnosed as having chronic osteomyelitis and then juvenile rheumatoid arthritis for 12 months. Pain disappeared soon after radiofrequency ablation, but the limited range of elbow motion was not improved with physical therapy. Imaging studies, including plain radiographs, computed tomography, and magnetic resonance imaging, revealed hypertrophic deformation of the bone and cartilage around the elbow joint. Despite repeated surgical debridement and manipulative arthrolysis, marked limitation of joint motion (30 degrees of flexion contracture and 110 degrees of further flexion) remained (Figure 2). Skeletal sequelae around the joint were not associated with the size of the osteoid osteoma.

## Discussion

Previous studies have shown that skeletal abnormalities, such as widening of the femoral neck, shortening of the femoral neck, coxa valga, increased femoral antetorsion, bowing of long bone, and overgrowth of long bone, may develop in children with osteoid osteoma (Table 3) [6-12]. Osteoid osteoma causes localized hyperemia, which may lead to overgrowth or angular deformity in the diaphysis or metaphyseal-diaphyseal junction of long bones [9]. In intra- and juxta-articular osteoid osteoma, the combined effects of synovitis and muscle contracture around the joint may induce more complex skeletal deformity [7]. In growing children with intra-articular osteoid osteoma, minimal periostitis around the growth plate might act as a catalyst for new bone formation, which causes skeletal hypertrophy at the femoral head-neck area [10]. Based on previous studies, however, most skeletal abnormalities induced by osteoid osteoma in skeletally immature patients are expected to disappear or improve after treatment for the nidus [6-12]. Even when skeletal deformities persist after symptom relief, especially in children younger than 5 years of age [9], the prognosis is reported to be benign. To the best of our knowledge, there have been no reported functional skeletal complications remaining after pain relief. However, our observations in the current study are different from previous authors in this respect. In our study, skeletal hypertrophy-related limitation of joint motion did not improve with physical therapy and required surgical interventions in 2 children with intra-/juxta-articular osteoid osteoma. Although signs of femoroacetabular impingement disappeared after osteochondroplasty through the surgical hip dislocation approach in one child, a markedly limited range of elbow motion persisted despite 2 subsequent surgeries and manipulative arthrolysis in the other child.

**Table 3:** **Skeletal abnormalities associated with osteoid osteoma in skeletally immature patients**

**Study**	**Case number**	**Age (yrs)**	**Skeletal abnormalities**	**Related symptoms**
Current study	6	6.2	Widening of the femoral neck, Coxa valga	N
		14.1	Hypertrophic deformation around the elbow	Elbow stiffness
		8.3	Widening of the femoral neck, Overgrowth (11 mm) of the femur	N
		13.5	Widening of the femoral neck, Coxa valga	N
		9.7	Widening of the femoral neck, Hypertrophy of the femoral head	Cam-type FAI
		5.1	Overgrowth (10 mm) of the tibia	N
Ponseti and Barta [11]	2	1.2	Varus deformity of the ankle joint	N
		2	Inward bowing of the tibia	N
Giustra and Freiberger [7]	2	8.5	Widening of the femoral neck, Flattened femoral head, Thickened acetabular rim	N
		13	Widening of femoral neck, Coxa valga, Enlarged femoral head	N
Norman and Dorfman [9]	6	2	Overgrowth medial half of the femur	N
		4.5	Overgrowth with lateral bowing of the radius	N
		1.3	Overgrowth (30 mm) with anterior bowing of the femur	N
		1	Overgrowth (38 mm) with medial bowing of the tibia	N
		5	Overgrowth (30 mm) of the phalanx	N
		2.5	Overgrowth of the ulna	NR
Kumar et al. [8]	3	9	Widening of the femoral neck	N
		11	Widening of the femoral neck	N
		12	Widening of the femoral neck	N
Cassar-Pullicino et al. [6]	1	6	Widening of the femoral neck	N
Xiao et al. [12]	1	9	Widening of the femoral neck, Enlarged femoral head	N
Pianta et al. [10]	1	16	Widening of the femoral neck	Cam-type FAI

FAI, femoroacetabular impingement; N, none; NR, no record.

This study clearly shows that osteoid osteoma in children has different clinical and radiological features depending on whether the nidus is located in/near the joint or not. The leading symptom of intra-/juxta-articular osteoid osteoma was painful limitation of joint motion secondary to synovitis, and this sign was present in 73% of children, whereas this symptom was not observed in any children with extra-articular osteoid osteoma. On the other hand, the classic symptoms of osteoid osteoma, nocturnal pain and the efficacy of NSAIDs for pain relief, were present in only 36% of children with intra-/juxta-articular osteoid osteoma compared with 70% of children with extra-articular osteoid osteoma, although the difference was not statistically significant. These different clinical manifestations are closely related to synovitis of the involved joint. Kawaguchi and colleagues [13] showed that osteoblasts in the nidus initiate arachidonic acid metabolism, which induces a variety of metabolic products that mediate or modulate inflammatory reactions. Kattapuram and colleagues [14] postulated that NSAIDs would be less effective in relieving pain in intra-articular osteoid osteoma due to secondary synovitis. Radiological findings were also different between the two types of osteoid osteoma. The typical plain radiographic feature of osteoid osteoma is a radiolucent or partially calcified nidus surrounded by a sclerotic margin [1], but in the current study, this finding was observed in only 36% of children with intra-/juxta-articular osteoid osteoma, whereas it was present in 78% of children with extra-articular osteoma. Intra-articular osteoid osteoma may appear as an absence or minimal degree of osteosclerosis with subtle periosteal reaction, and the nidus may appear as regional osteoporosis, especially when it is located around the hip joint [6,15].

There are some limitations of this study that should be considered. First, the number of patients, especially with intra-/juxta-articular osteoid osteoma, was small. We suspect that prolonged hyperemia and synovitis due to diagnostic delay might have caused the severe hypertrophic deformation around the joint that led to permanent sequelae, but we could not validate this relationship with a statistical analysis because of the small number of cases. Second, we included patients who were followed for at least 2 years, but some children did not reach skeletal maturity at the final follow-up evaluation. Remodeling of skeletal deformities and thus change in shape would be expected by physeal and epiphyseal growth until reaching skeletal maturity. Third, the follow-up interval was not regular due to the retrospective nature of the study, which limited our detailed analysis of the responses to the treatment and remodeling of the skeletal deformities. Fourth, the majority of osteoid osteomas were located in the femur (n = 20) and the tibia (n = 8), so generalization of the results in the current study to all skeletal locations may be biased.

## Conclusions

Our observations suggest that the clinical and radiological features of intra-/juxta-articular osteoid osteoma in growing children differ from those of extra-articular osteoma. Skeletal abnormalities are found mainly in intra-/juxta-articular osteoid osteoma, and skeletal hypertrophy around the joint may lead to permanent sequelae, such as limited range of joint motion. Our study suggests that understanding the unusual clinical and radiological features of intra-/juxta-articular osteoid osteoma and thus early therapeutic intervention may prevent potential skeletal complications in skeletally immature patients.

## Abbreviation

NSAID: Nonsteroidal anti-inflammatory drug
